# Lymph node mapping-based optimal bowel-resection margin and central radicality in colon cancer surgery: an international, prospective, observational cohort study

**DOI:** 10.1016/j.esmogo.2025.100231

**Published:** 2025-08-27

**Authors:** H. Ueno, N.K. Kim, J.C. Kim, P. Tsarkov, W. Hohenberger, R. Grützmann, N.E Samalavičius, A. Dulskas, J.-T. Liang, P. Quirke, N. West, A. Shiomi, M. Ito, M. Shiozawa, K. Komori, K. Matsuda, Y. Kinugasa, T. Sato, K. Yamada, Y. Hashiguchi, H. Ozawa, Y. Kanemitsu, T. Kusumi, H. Ike, Y. Takii, H. Matsuoka, Y. Toiyama, J. Watanabe, A. Ishibe, H. Sonoda, K. Koda, F. Fujita, M. Ohue, M. Itabashi, M. Taketsuna, S. Higashide, Y. Ajioka, K. Sugihara

**Affiliations:** 1 Department of Surgery, National Defense Medical College, Saitama, Japan; 2 Department of Surgery, Yonsei University College of Medicine, Seoul, Republic of Korea; 3 Division of Colon and Rectal Surgery, Department of Surgery, University of Ulsan College of Medicine, Asan Medical Center, Seoul, Republic of Korea; 4 Department of Surgery, Faculty of Preventive Medicine, Clinic of Colorectal and Minimally Invasive Surgery, Sechenov First Moscow State Medical University, Moscow, Russia; 5 Department of Surgery, University Hospital Erlangen, Friedrich-Alexander-University of Erlangen-Nuremberg, Erlangen, Germany; 6 Center of General Surgery, Republican Vilnius University Hospital, Vilnius, Lithuania; 7 Clinic of Internal Diseases and Family Medicine, Faculty of Medicine, Institute of Clinical Medicine, Vilnius University, Vilnius, Lithuania; 8 Department of General and Abdominal Surgery and Oncology, National Cancer Institute, Vilnius, Lithuania; 9 Division of Colorectal Surgery, Department of Surgery, National Taiwan University Hospital and College of Medicine, Taipei, Taiwan; 10 Pathology and Data Analytics, Leeds Institute of Medical Research at St. James’s, University of Leeds, Leeds, UK; 11 Division of Colorectal Surgery, Shizuoka Cancer Center Hospital, Shizuoka, Japan; 12 Colorectal and Pelvic Surgery Division, Department of Surgical Oncology, National Cancer Center Hospital East, Chiba, Japan; 13 Department of Gastrointestinal Surgery, Kanagawa Cancer Center, Kanagawa, Japan; 14 Department of Gastroenterological Surgery, Aichi Cancer Center Hospital, Aichi, Japan; 15 Second Department of Surgery, School of Medicine, Wakayama Medical University, Wakayama, Japan; 16 Department of Gastrointestinal Surgery, Institute of Science Tokyo, Tokyo, Japan; 17 Department of Surgery, Yamagata Prefectural Central Hospital, Yamagata, Japan; 18 Department of Gastroenterological Surgery, Coloproctology Center, Takano Hospital, Kumamoto, Japan; 19 Department of Surgery, Teikyo University School of Medicine, Tokyo, Japan; 20 Department of Surgery, Tochigi Cancer Center, Tochigi, Japan; 21 Department of Colorectal Surgery, National Cancer Center Hospital, Tokyo, Japan; 22 Department of Surgery, Keiyukai Sappro Hospital, Hokkaido, Japan; 23 Department of Surgery, Saisei-kai Yokohama-shi Nanbu Hospital, Kanagawa, Japan; 24 Department of Surgery, Niigata Cancer Center Hospital, Niigata, Japan; 25 Department of Surgery, Kyorin University School of Medicine, Tokyo, Japan; 26 Department of Gastrointestinal and Pediatric Surgery, Mie University Graduate School of Medicine, Mie, Japan; 27 Department of Surgery, Gastroenterological Center, Yokohama City University Medical Center, Kanagawa, Japan; 28 Department of Gastroenterological Surgery, Yokohama City University Graduate School of Medicine, Kanagawa, Japan; 29 Department of Surgery, Shiga University of Medical Science, Shiga, Japan; 30 Department of Surgery, Teikyo University Chiba Medical Center, Chiba, Japan; 31 Department of Surgery, Kurume University School of Medicine, Fukuoka, Japan; 32 Department of Gastroenterological Surgery, Osaka International Cancer Institute, Osaka, Japan; 33 Department of Surgery, Institute of Gastroenterology, Tokyo Women’s Medical University, Tokyo, Japan; 34 Translational Research Center for Medical Innovation, Foundation for Biomedical Research and Innovation, Hyogo, Japan; 35 Division of Molecular and Diagnostic Pathology, Graduate School of Medical and Dental Science, Niigata University, Niigata, Japan; 36 Institute of Science Tokyo, Tokyo, Japan

**Keywords:** colon cancer, bowel-resection margin, central radicality, D3 lymph node dissection, complete mesocolic excision, lymph node mapping

## Abstract

**Background:**

Substantial variations in the extent of lymphadenectomy are acknowledged internationally in colon cancer surgery because essential data for standardization, including the anatomical distribution of metastatic lymph nodes (LN), are lacking.

**Materials and methods:**

Pre-specified LN mappings based on *in vivo* bowel measurements were conducted for stages I-III colon cancer patients treated at 31 leading hospitals in six countries. The extent of lymphadenectomy was classified from levels A (pericolic) to C (central LNs) according to the pre-specified anatomical landmarks. The primary outcome was the extent of pericolic lymphatic spread and the incidence of metastasis in central LNs, and secondary ones included the real-world status of central radicality and its association with short-term outcomes.

**Results:**

Among 3647 patients, pericolic spread beyond 10 cm (0.2%) and absence of feeding arteries supplying the bowel within 10 cm from the primary tumor (0.3%) were rare, irrespective of nationality. The incidence of metastasis in central LNs was ∼3% (range: 0.2% in T1 to 7% in T4 tumors) and was lower in tumors located at the splenic flexure (0.5%). The proportion of patients with level C radicality was ∼76%, which was statistically significantly associated with T stage only in one country. A higher radicality level conferred no adverse impact on either the incidence of Clavien–Dindo grade ≥III or 30-day mortality.

**Conclusions:**

The ‘10-cm rule’ could be an international criterion for determining the bowel-resection margin. Central lymphadenectomy is feasible internationally, though the indication should be selective, not routine, depending on the stage and location of the primary tumor.

## Highlights

• This is the first multi-national study for LN mapping with Western and Eastern populations of colon cancer patients.
• There is no ethnic difference in the anatomical distribution of metastatic LNs and feeding arteries.
• The ‘10-cm rule’ could be an optimal international criterion for determining the bowel-resection margin.
• Complete removal of intermediate LNs is imperative even for T1 or T2 tumors considering the incidence of metastatic tumors.
• The indication of central LN dissection should be tailored, most preferably by T stage and tumor location.

## Graphical abstract

ICA, ileocolic artery; IMA, inferior mesenteric artery; LCA, left colic artery; MCA, middle colic artery; RCA, right colic artery; SA, sigmoid artery; SMA, superior mesenteric artery; SMV, superior mesenteric vein.

**Figure:**
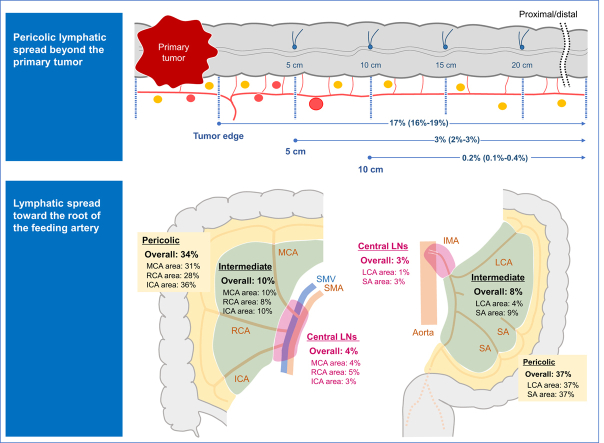

## Introduction

In the era of advanced molecular targeted therapy, the *en bloc* removal of the primary tumor and regional lymph nodes (LN), which potentially harbor metastatic tumor cells, remains the cardinal first step to cure malignant epithelial gastrointestinal tumors. International consensus criteria on the area of ‘regional’ LN that should be removed in routine surgical practice constitutes an important unsolved challenge for optimizing and standardizing lymphadenectomy for colon cancer. Considerable international variation in the definition of ‘regional’ pericolic nodes confers uncertainty regarding the optimal bowel-resection margin.^1^^,^^2^ Furthermore, the standard central radicality in colon cancer surgery, that is, the anatomical area of LNs in the lymphovascular networks from the primary tumor toward the superior mesenteric artery (SMA) or inferior mesenteric artery (IMA) that should be routinely excised, has not been unified internationally.

In 1977, the concept of D3 dissection was first documented in the *Japanese Classification of Cancer of the Colon and Rectum* staging manual,^3^ where the proportion of patients receiving this procedure in Japan had reached 75% among those with stages II and III colorectal cancer in 2010.^4^ D3 dissection was favorably accepted in Asian countries, including the Republic of Korea, People’s Republic of China, and Taiwan.^5^^,^^6^ During the 2010s, with the advent of complete mesocolic excision (CME) proposed by Hohenberger et al.,^7^ a global trend had emerged wherein extended lymphadenectomy was considered a hallmark of high-quality surgery.^1^^,^^8^ Recent meta-analyses demonstrated survival benefits of the D3/CME surgery.^9^^,^^10^ However, owing to considerable bias in previous studies, there is still limited evidence of the long-term oncological benefit of extended lymphadenectomy, and the jury is still out on whether routine D3/CME is justified in colon cancer surgery.^11^, ^12^, ^13^, ^14^, ^15^

Uncertainty regarding the value of extended lymphadenectomy may be attributed substantially to the lack of conclusive data on the actual anatomical distribution of metastatic LNs that had been estimated with internationally unified methods. First, the actual incidence of tumor spread in the area that can be exclusively removed by extended lymphadenectomy remains unknown, which confers uncertainty about the possible magnitude of oncological benefit that can be gained from such techniques. Second, the possibility of ethnic differences in lymphovascular anatomy that could lead to different anatomical distribution of metastatic LNs remains unascertained. Such information is essential to determine whether the extent of lymphadenectomy in colon cancer surgery can be standardized globally.

The T-REX study is a prospective observational cohort study that involves international data collection on the anatomical distribution of metastatic LNs and the clinical outcomes of lymphadenectomy with the pre-specified LN categorization and the extent of lymphadenectomy according to anatomical landmarks.^16^ This study was conducted with an aim to establish fundamental data for implementing evidence-based recommendations on the extent of bowel resection and appropriate central lymphadenectomy based on an in-depth analysis of LN and vascular mapping to define ‘regional’ LN in colon cancer surgery.

## Methods

### Study design and participants

Patients were eligible for recruitment if diagnosed with preoperative stage I, II, or III colon cancer and would receive potentially curative surgery between 30 May 2013 and 31 December 2018.^16^ The inclusion and exclusion criteria are shown in Supplementary Figure S1, available at https://doi.org/10.1016/j.esmogo.2025.100231. The patient registration started antecedently in 24 institutions of the Japanese Society for Cancer of the Colon and Rectum (JSCCR) on 30 May 2013, and then in seven leading hospitals in Republic of Korea, Russia, Lithuania, Germany, and Taiwan on 22 January 2015 (Supplementary Table S1, available at https://doi.org/10.1016/j.esmogo.2025.100231). After the exclusion of 49 ineligible patients, data from 3647 patients were included for analyses (Supplementary Figure S1, available at https://doi.org/10.1016/j.esmogo.2025.100231). The baseline characteristics of the study population are presented in Supplementary Table S2, available at https://doi.org/10.1016/j.esmogo.2025.100231. The mean number of LN examined per patient was 31.5 and ranged from 29.4 in Lithuania to 50.8 in Russia.

The study protocol, including the final version of the subject information and consent forms, were approved by the Ethics Committee of the JSCCR and the Investigational Review Board of each participating center. All participants provided written informed consent in each center and the study was undertaken in accordance with the Declaration of Helsinki. The study design has previously been published,^16^ and the study was registered with ClinicalTrials.gov (NCT02938481).

### Procedures

Retrieved LNs were grouped as pericolic, intermediate, or central (main or D3)^17^ based on the LN grouping system of the *Japanese Classification of Colorectal Carcinoma* issued by the JSCCR.^16^^,^^18^ Central LNs were defined as those along the SMA or superior mesenteric vein (SMV) at the root of the colic artery for right-sided colon cancers and those along the IMA proximal to the origin of the left colic artery (LCA) for left-sided colon cancers.^18^ The anatomical location of the feeding artery and pericolic LNs were categorized based on pre-planned *in vivo* bowel measurements. Specifically, the distance from the closest tumor edge was measured intraoperatively in the natural state without external tension and the points at 5, 10, 15, and 20 cm from the proximal and distal edge of the tumor were respectively marked on the bowel wall (the colon or terminal ileum) with serosal sutures, yielding a total of 11 pericolic segments. The measurement points on the terminal ileum were used to determine the distance from the primary tumor at the cecum or proximal ascending colon. On surgical specimens, the primary feeding artery was identified, and its location was recorded as the pericolic segment that the feeding artery supplied. Similarly, pericolic LNs were categorized based on the bowel segmentation. All LNs harvested from the resected surgical specimens were pathologically examined at each institution. Mesocolic tumor nodules without pathological evidence of LN structure were treated as metastatic LNs, irrespective of their size and contour morphology.^19^

The level of central radicality was recorded with the original categorization of levels A to C according to pre-specified anatomical landmarks (Supplementary Figure S2, available at https://doi.org/10.1016/j.esmogo.2025.100231).^16^ Level C designated central radicality where the central LNs had been dissected.

### Outcomes

All baseline and investigative data were collected via the electronic data capture system (eClinical Base; Translational Research Center for Medical Innovation, Japan).^16^ The primary outcome of this study was anatomical distribution of metastatic LNs with special interest in the location of the most distant metastatic pericolic LNs and the incidence of metastasis in central LNs. To calculate the incidence of metastatic LNs in specific pericolic segments, we counted patients with metastases in either or both segments proximal and distal to the primary tumor. Sub-group analyses for primary outcome were carried out based on various essential clinicopathological factors, including pathological T (pT) stage in the TNM system stage, the primary feeding artery, the location of the primary feeding artery, location of the primary tumor, and populations. Secondary outcomes included the real-world status of central radicality in colon cancer surgery adopted in six countries and its association with short-term clinical outcomes, such as operation time, blood loss, the Clavien–Dindo grade, and 30-day mortality.

### Statistical analysis

The chi-square test was used to assess differences in the location of the primary artery distributed into the pericolic region, location of the most distant metastatic pericolic LN, incidence of metastasis in the pericolic, intermediate, and central LNs, and central radicality. The Jonckheere–Terpstra trend test was used to assess association between the central radicality and operation time or blood loss. In addition, Cochran–Armitage trend test was used to assess association between the central radicality and proportion of patients with Clavien–Dindo grade ≥III or 30-day mortality. Statistical analyses were carried out using SAS Version 9.4 (SAS Institute Inc., Cary, NC).

### Role of the Funding source

The JSCCR was responsible for the management of the T-REX study including study design, protocol development, data collection, data interpretation, and the writing of the report. The Translational Research Center for Medical Innovation supported the data management for this study with eClinical Base and the data analysis with funds from the Ministry of Health, Labor and Welfare in Japan/Japan Agency for Medical Research and Development and Foundation for Biomedical Research and Innovation at Kobe.

## Results

The total number of LN examined in this study was 114 876. The mean number of pericolic LNs retrieved per patient was 20.9, and the country-specific numbers ranged from 17.7 in Taiwan to 29.7 in Russia (Supplementary Table S2, available at https://doi.org/10.1016/j.esmogo.2025.100231). The number of pericolic LNs retrieved was positively correlated with the pT stage (range: 16.8 for pT1 to 24.0 for pT4; Supplementary Table S3, available at https://doi.org/10.1016/j.esmogo.2025.100231), was the highest in the primary tumor segment (mean: 5.2), and decreased according to the distance from the primary tumor (Supplementary Figure S3, available at https://doi.org/10.1016/j.esmogo.2025.100231).

Feeding artery mapping showed that the primary feeding artery supplied the primary tumor segment in 58% of patients (Supplementary Figure S3, available at https://doi.org/10.1016/j.esmogo.2025.100231). Patients without a feeding artery to the pericolic region within 10 cm from the primary tumor margin were rare [*n* = 11 (0.3%)], regardless of nationality (Supplementary Table S4, available at https://doi.org/10.1016/j.esmogo.2025.100231). Exceptionally, the proportion of patients with primary feeding artery supply to the pericolic region beyond 10 cm from the primary tumor margin increased to 2% (4 out of 192 patients) in those with primary tumors at the splenic flexure.

**Figure 1:**
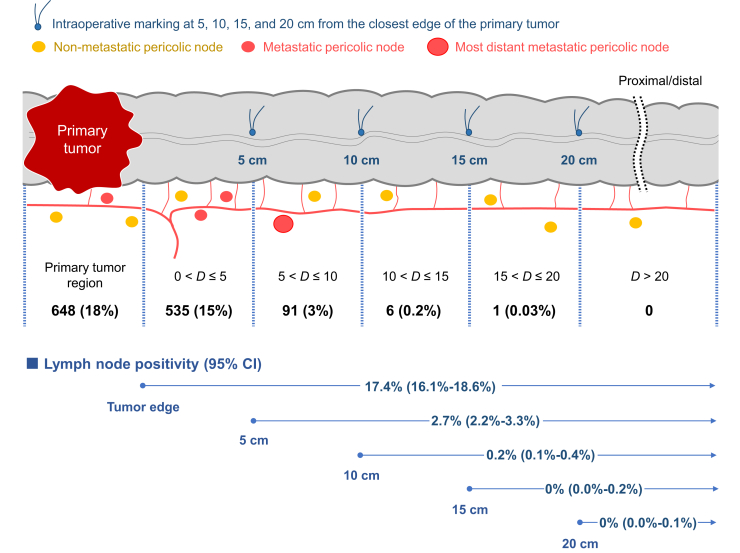
**Location of the most distant metastatic pericolic lymph node.** The pericolic region was divided into six segments according to the *in vivo* marking stitches made at 5, 10, 15, and 20 cm from the primary tumor. The figures in the upper row demonstrate the number (%) of patients whose most distant metastatic pericolic lymph node was located in each segment, irrespective of distal and proximal location from the primary tumor. Note that the primary tumor region was the most common segment with the most distant pericolic lymph node involvement, and the incidence decreased as the distance from the primary tumor increased. As shown in the lower row, the analysis for lymph node positivity indicates that the 10-cm resection margin of the bowel would be associated with only a 0.2% chance of residual metastatic pericolic nodes. CI, confidence interval; *D*, distance from the closest primary tumor edge (cm).

The incidence of pericolic LN metastasis was the highest in the primary tumor segment and it decreased according to the distance from the primary tumor (Supplementary Figure S3, available at https://doi.org/10.1016/j.esmogo.2025.100231). With regard to the locations of the primary tumor or the primary feeding artery, no significant difference was found in the anatomical distribution of the most distant metastatic pericolic LNs (Supplementary Table S5, available at https://doi.org/10.1016/j.esmogo.2025.100231). Patients with metastatic pericolic LNs beyond 10 cm from the primary tumor margin were rare (*n* = 7 overall) in any country, as reported from four institutions in Japan (5 cases), an institution in the Republic of Korea (1 case), and an institution in Russia (1 case). Among these patients, five had massive LN metastasis, such as pN2b, or central LN involvement (Supplementary Table S6, available at https://doi.org/10.1016/j.esmogo.2025.100231). The LN positivity beyond 10 cm from the primary tumor margin was 0.2% (95% CI 0.1% to 0.4%; Figure 1). However, with a 5 cm cut-off distance from the primary tumor margin, LN positivity beyond the cut-off value increased to 2.7% (95% CI 2.2% to 3.3%). Pericolic LN metastasis beyond 5 cm was, however, observed only in five cases (0.8%) in patients with pT1 tumors (Supplementary Table S3, available at https://doi.org/10.1016/j.esmogo.2025.100231).

**Figure 2:**
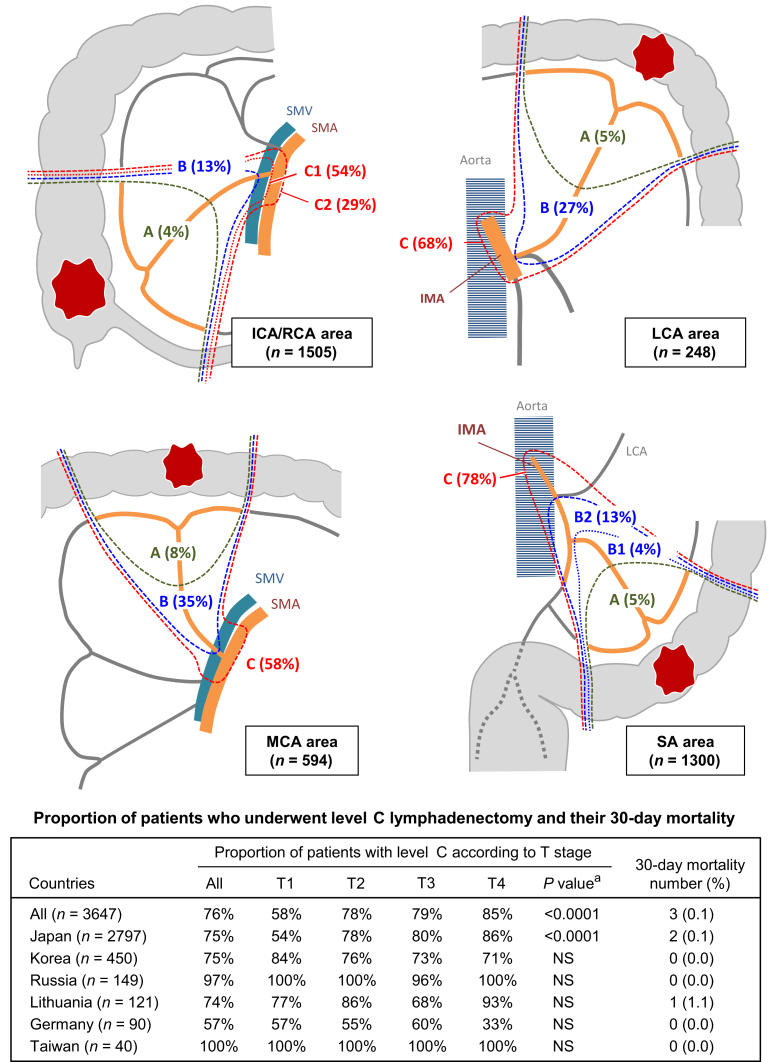
**Real-world status of central radicality in specialist institutions of six countries.** The level of central radicality was prospectively recorded with the original categorization of levels A to C according to pre-specified anatomical landmarks (Supplementary Figure S2, available at https://doi.org/10.1016/j.esmogo.2025.100231). Colectomy with level C central radicality was carried out in a total of 2767 patients (76%). The incidence differed depending on tumor location. Compared with tumors located at the MCA area, tumors located at the ICA/RCA and SA area had higher central radicality. The 30-day mortality was observed only in 3 out of 2767 patients (0.1%). Only in Japanese patients, participants who underwent surgery with level C central radicality showed a significant association with the pathological T stage. ICA, ileocolic artery; IMA, inferior mesenteric artery; LCA, left colic artery; MCA, middle colic artery; NS, not significant; PT, primary tumor; RCA, right colic artery; SA, sigmoid artery; SMA, superior mesenteric artery; SMV, superior mesenteric vein. ^a^Chi-square test.

Level A, B, and C lymphadenectomy was carried out in 5%, 19%, and 76% of the study population, respectively (Supplementary Table S2, available at https://doi.org/10.1016/j.esmogo.2025.100231). Level C lymphadenectomy was carried out in ∼80% of tumors with the ileocolic artery/right colic artery (ICA/RCA) or the sigmoid artery as the primary feeding artery, but in only 68% and 58% of tumors at the LCA area and middle colic artery (MCA) area, respectively (Figure 2). This reduction in tendency for level C lymphadenectomy in the latter groups was similar for the sub-population of pT4 tumors (Supplementary Table S7, available at https://doi.org/10.1016/j.esmogo.2025.100231).

The country-specific incidence of level C lymphadenectomy varied, ranging from 57% for Germany to ∼100% for Russia or Taiwan. The proportion of patients who underwent level C lymphadenectomy was significantly associated with T stage in Japan, whereas no association between the T stage and central radicality was detected for other countries (Figure 2).

**Figure 3:**
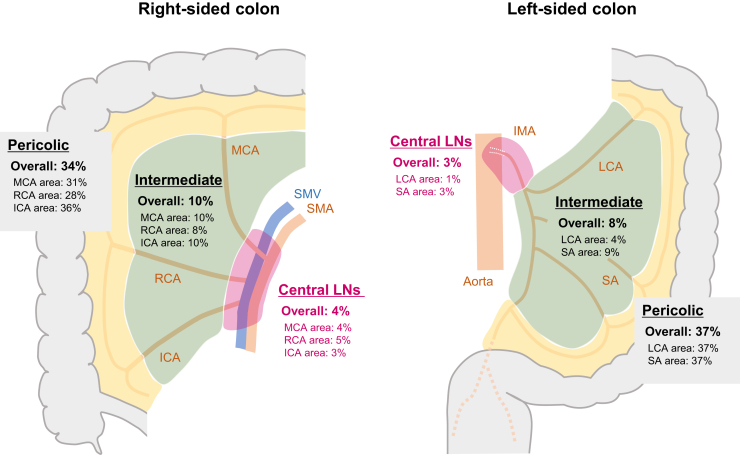
**The incidence of metastasis in pericolic, intermediate, and central lymph nodes stratified by the first feeding artery of the tumor.** The respective values were calculated with the total number of patients in the analysis set (*n* = 3647) as the denominator. Furthermore, 3%-5% of patients with stage I-III colon cancer had metastasis to central LNs, irrespective of the tumor location, except for those with tumors in the LCA area with a corresponding incidence of only ∼1%. ICA, ileocolic artery; IMA, inferior mesenteric artery; LCA, left colic artery; LN, lymph node; MCA, middle colic artery; RCA, right colic artery; SA, sigmoid artery; SMA, superior mesenteric artery; SMV, superior mesenteric vein.

In the analysis dataset, 3% of patients had metastasis in central LNs (4% and 3% in right- and left-sided colonic tumors, respectively; Figure 3) and showed a wide range from 0.2% to 7%, depending on the T stage (Table 1). The incidences of metastasis in intermediate and central LNs were relatively low (4% and 1%, respectively) in tumors located at the LCA area. In addition, as compared with the incidences of metastases in intermediate and central LN tumors located at the non-flexural site (9% and 3%, respectively), the incidence was lower in tumors located at the splenic flexure (0.5% and 0.5%, respectively) and, conversely, was relatively higher in tumors located at the hepatic flexure (12% and 6%, respectively; Table 1).

**Table 1:** Incidence of metastasis in the pericolic, intermediate, and central lymph nodes

	Overall		Pericolic LNs		Intermediate LNs		Central LNs	
	*n* (%)	*P* value	*n* (%)	*P* value	*n* (%)	*P* value	*n* (%)	*P* value
Overall	1383 (38)	—	1281 (35)	—	330 (9)	—	114 (3)	—
Tumor location (categorization 1)^a^		0.42		0.10		0.0004		0.58
C (*n* = 470)	179 (38)		165 (35)		58 (12)		16 (3)	
A (*n* = 1115)	417 (37)		391 (35)		86 (8)		37 (3)	
T (*n* = 539)	188 (35)		163 (30)		57 (11)		20 (4)	
D (*n* = 244)	92 (38)		91 (37)		8 (3)		4 (2)	
S (*n* = 1279)	507 (40)		471 (37)		121 (10)		37 (3)	
Tumor location (categorization 2)		0.29		0.12		<0.0001		0.0016
Hepatic flexure (*n* = 364)	132 (36)		111 (31)		42 (12)		21 (6)	
Splenic flexure (*n* = 192)	64 (33)		64 (33)		1 (0.5)		1 (0.5)	
Non-flexure site (*n* = 3091)	1187 (38)		1106 (36)		287 (9)		92 (3)	
Location of the primary feeding artery^b^		0.043		0.11		0.85		0.76
PT region (*n* = 2117)	819 (39)		761 (36)		199 (9)		63 (3)	
≤5 cm from the PT (*n* = 1298)	497 (38)		456 (35)		112 (9)		42 (3)	
5-10 cm from the PT (*n* = 196)	56 (29)		54 (28)		16 (8)		8 (4)	
>10 cm from the PT (*n* = 11)	5 (46)		5 (46)		1 (9)		0	
T stage		<0.0001		<0.0001		<0.0001		<0.0001
T1 (*n* = 660)	79 (12)		74 (11)		12 (2)		1 (0.2)	
T2 (*n* = 534)	134 (25)		120 (23)		24 (5)		7 (1)	
T3 (*n* = 1867)	780 (42)		718 (39)		190 (10)		63 (3)	
T4 (*n* = 586)	390 (67)		369 (63)		104 (18)		43 (7)	
Countries		0.71		0.52		0.049		<0.0001
Japan (*n* = 2797)	1069 (38)		997 (36)		240 (9)		56 (2)	
Korea (*n* = 450)	166 (37)		145 (32)		45 (10)		41 (9)	
Germany (*n* = 90)	29 (32)		27 (30)		10 (11)		1 (1)	
Russia (*n* = 149)	62 (42)		58 (39)		16 (11)		7 (5)	
Lithuania (*n* = 121)	42 (35)		40 (33)		10 (8)		6 (5)	
Taiwan (*n* = 40)	15 (38)		14 (35)		9 (23)		3 (8)	

LN, lymph node; PT, primary tumor.

a C, A, T, D, and S represent the cecum, ascending colon, transverse colon, descending colon, and sigmoid colon, respectively.

b Data from a total of 3622 patients in the analysis dataset were analyzed after excluding 25 patients whose data on the location of the primary feeding artery were missing.

Regarding short-term clinical outcomes, the blood-loss volume was greater in patients with a higher central radicality level, though an increased radicality level had no adverse impact on operation time, the incidence of Clavien–Dindo grade ≥III, or 30-day mortality (Table 2). In the sub-group analyses, an increased level of radicality was significantly associated with longer operation time in patients with primary tumors in the ICA/RCA and MCA areas. The 30-day post-operative mortality with level C lymphadenectomy was three (0.1%; Japan, *n* = 2 and Lithuania, *n* = 1; Figure 2).

**Table 2:** Short-term outcomes stratified by the extent of central radicality

Primary feeding artery	Central radicality^a^	Operation time		Blood loss		Clavien–Dindo grade ≥III		30-day mortality	
		Minutes, median (IQR)	*P* value^b^	ml, median (IQR)	*P* value^b^	*n* (%)	*P* value^c^	*n* (%)	*P* value^c^
**Overall**	A (*n* = 191)	192 (158-239)	0.50	30 (10-80)	0.0078^d^	6 (3)	0.52	1 (0.5)	0.0018^e^
	B (*n* = 689)	192 (155-237)		23 (10-55)		24 (4)		7 (1)	
	C (*n* = 2767)	192 (154-238)		30 (10-72)		80 (3)		3 (0.1)	
***ICA/RCA***	A (*n* = 65)	181 (148-215)	<0.0001^d^	25 (10-50)	<0.0001^d^	1 (2)	0.32	0	0.90
	B (*n* = 199)	173 (142-208)		19 (8-58)		8 (4)		1 (0.5)	
	C1 (*n* = 805)	173 (139-212)		20 (10-50)		17 (2)		0	
	C2 (*n* = 436)	203 (169-247)		50 (20-100)		8 (2)		1 (0.2)	
***MCA***	A (*n* = 46)	200 (172-281)	0.021^d^	40 (15-83)	0.077	3 (7)	0.45	1 (2)	0.018^e^
	B (*n* = 206)	211 (170-249)		35 (13-74)		10 (5)		3 (2)	
	C (*n* = 342)	230 (180-268)		41 (15-110)		14 (4)		0	
***LCA***	A (*n* = 13)	228 (215-248)	0.32	80 (30-101)	0.26	1 (8)	0.52	0	0.52
	B (*n* = 66)	213 (168-254)		28 (17-50)		2 (3)		0	
	C (*n* = 169)	207 (165-251)		50 (10-100)		5 (3)		1 (0.6)	
***SA***	A (*n* = 67)	194 (160-220)	0.63	10 (0-80)	0.074	1 (2)	0.12	0	0.31
	B1 (*n* = 45)	211 (162-252)		50 (20-100)		0		0	
	B2 (*n* = 173)	187 (153-227)		12 (5-31)		4 (2)		3 (2)	
	C (*n* = 1015)	190 (153-235)		20 (8-58)		36 (4)		1 (0.1)	

ICA, ileocolic artery; IQR, interquartile range; LCA, left colic artery; MCA, middle colic artery; RCA, right colic artery; SA, sigmoid artery.

a The level of central radicality was recorded with the original categorization of levels A to C according to pre-specified anatomical landmarks (Supplementary Figure S2, available at https://doi.org/10.1016/j.esmogo.2025.100231).

b Jonckheere–Terpstra trend test.

c Cochran–Armitage trend test.

d A significant trend toward greater operation time or blood-loss volume with increasing degree of central radicality.

e A significant trend toward fewer chances of 30-day mortality with increasing degree of central radicality.

## Discussion

In this large international cohort study, the actual status of the anatomical distribution of metastatic LNs and feeding artery was reasonably similar among different countries. This finding supports the possibility of defining ‘regional’ LN and thereby standardizing the extent of lymphadenectomy in routine colon cancer surgery. Specifically, our prospective in-depth LN and vascular mapping data provides insight into the bowel-resection margin and the extent of central radicality.

Pericolic lymphatic spread beyond 10 cm was extremely rare (0.2%), irrespective of the distribution of the primary feeding artery or tumor location. This result was similar to that of a previous nationwide cohort study in Japan, in which metastatic pericolic nodes beyond 10 cm from the primary tumor margin were observed only in 0.1% in patients with stage I-III colon cancer.^20^ Both studies, however, indicate that metastatic pericolic nodes would remain unexcised, and thus cause recurrence, in at least 3% of patients if the bowel is resected with a 5-cm margin. These results indicate that the ‘10-cm rule’ may be an oncologically appropriate standard bowel-resection margin.^20^

Patients without feeding arteries within 10 cm from the primary tumor were exceptionally rare (0.3%), regardless of nationality. This figure indicates that the ‘10-cm rule’ ensures *en bloc* excision of the lymphovascular network from the primary tumor toward the origin of the feeding artery that is at risk of harboring metastatic tumor cells. The only exception was tumors located at the splenic flexure, where a wider bowel resection is needed, up to 15 cm from the primary tumor (Supplementary Table S4, available at https://doi.org/10.1016/j.esmogo.2025.100231), to implement *en bloc* excision of the tumor-bearing segment and the lymphatics draining along the feeding artery in ∼2% of the population.

The literature has often used the terminologies D3 dissection and CME interchangeably.^6^^,^^21^ In the original Hohenberger’s CME technique, extra-mesenteric LNs such as those over the head of the pancreas and those along the gastroepiploic arcade were resected for cancer located at the transverse colon^7^^,^^22^; however, the removal of central, rather than extra-mesenteric, LNs was recently regarded as the principal requirement for CME in terms of the central radicality,^15^^,^^17^ exactly as in a D3 dissection.^18^ Our LN mapping clarified that central LNs are involved by metastatic tumor in ∼3% of patients with stage I-III disease.

Extended lymphadenectomy characterized by the removal of central LNs is highly valued in the literature recently,^9^^,^^10^^,^^23^ and it was shown that central LN metastasis is not a sign of systemic disease per se and that the 5-year overall survival rate of those with such disease exceeds 70% if the patient has no residual disease.^24^ Nevertheless, its routine execution may not be justified based on the risk–benefit equation,^14^ especially when we consider the relatively small chance of central LN metastasis that suggests a limited survival benefit brought about by routine extended lymphadenectomy.^11^^,^^13^ In this regard, this study provides some fundamental data that are useful to optimize the indication of extended lymphadenectomy. First, the risk of central LN metastasis was effectively stratified by the T stage. The incidence of central LN metastasis was only 0.2% for T1 and 1% for T2, indicating that routine extended lymphadenectomy is not necessarily needed for these populations, although the complete dissection of intermediate LNs is imperative even for T1 or T2 tumors considering 2% to 5% of patients with such disease are harboring metastatic tumors in intermediate LNs. Second, this study has clarified the incidences of metastasis in the intermediate and central LNs were rare (<1%) in tumors located at the splenic flexure, presumably due to the sparse vascular distribution into the splenic flexure.

Internationally unified anatomy-based grading of lymphadenectomy is a prerequisite to estimate the value of lymphadenectomy more specifically and this study draws attention to the ambiguity of ‘non-CME’ procedure used in the literature. For example, Gao et al. reported a beneficial effect of CME for non-metastatic colon cancer patients with the significant superiority of 3-year local recurrence-free survival rate by 10% as compared with ‘non-CME’ based on a prospective study.^25^ Bertelsen et al. reported a significant survival superiority of CME compared with ‘non-CME’ in patients with stage I and II disease by >10% in a population-based study with propensity score matching system.^26^ Given the estimated incidence of central LN metastasis clarified in this study, survival benefits observed in the CME group are not satisfactorily explained only by the effect of the removal of central LNs. More specifically, it is highly supposed that ‘non-CME’ was a technique that left not only central LNs, but a part of ‘regional’ intermediate LNs unexcised, which is entirely different from ‘D2’ dissection that requires complete removal of intermediate LNs.^18^ We emphasize that D2 dissection is an imperative surgical procedure even for T1 or T2 tumors based on our data on the incidence of metastasis in intermediate LNs.

In some meta-analyses, CME was associated with increased incidence of vascular injury.^9^ In the present study, blood-loss volume was slightly greater in patients with a higher central radicality, but increased radicality had no adverse impact on Clavien–Dindo grade ≥III nor 30-day mortality. In addition, the number of patients who died within 30 days after operation with level C radicality was only three (0.1%). These results seem consistent with the results of a randomized controlled trial (RCT) reported from China.^27^ Di Buono et al. recently reported that laparoscopic CME was comparable with non-CME in terms of post-operative complication in an Italian population, whose median body mass index (BMI) was 25.5, as the result of RCT.^28^ Our study population included patients with a greater BMI, such as those in Lithuania, Germany, and Russia, and the data suggest that central LN dissection could be feasible globally. Nevertheless, we should underscore that all operations were carried out by experienced colorectal cancer surgeons in our study, because some reports, including a large population-based study in Denmark^29^ and some meta-analyses,^9^^,^^30^^,^^31^ show that CME was associated with more intraoperative organ injuries and post-operative morbidity.

As was expected, this study disclosed a significant ethnic difference in physique of the patients, especially between patients in Asian and European countries.^2^ Nevertheless, on top of the LN positivity and LN ratio, the variability regarding the incidence of the primary feeding artery or metastatic pericolic nodes located >10 cm from the primary tumor and the incidence of pericolic or intermediate LN metastasis were quite small by countries. Despite some degree of difference in the incidence of positive central LNs among countries, possibly due to different anatomical boundary definitions between intermediate and central LNs, we concluded that the anatomical distribution of the primary feeding artery or metastatic LNs, as well as the biological tumor aggressiveness in terms of lymphatic metastasis, is reasonably similar among countries.

This study has several limitations. First, this was an observational cohort study, not an RCT. Therefore, we cannot definitively evaluate the impact of implementation of the ‘10-cm rule’ or that of central LN dissection as compared with no implementations. The data obtained in this study, however, constitutes the best current data and surely provides compelling evidence to standardized colon cancer surgery. RCTs, though ideal, may be practically difficult to establish,^12^ considering the enormous number of patients needed for statistical power to demonstrate the survival benefit of the removal of pericolic nodes located >10 cm from the primary tumor margin or central nodes. Second, the proportion of patients from Western countries was relatively small in this study; thus, future studies are warranted to confirm the conclusion of this study in other populations. However, the design of this study was strengthened by the fact that all participating institutions were specialist institutions with high levels of surgical quality, and the LN data were prospectively collected based on pre-specified methods, which may be confirmed by the large number of LNs evaluated in this study. Third, our grading system for the extent of central radicality did not include extra-mesenteric LNs, though we were aware that dissection of those LNs was needed in a small proportion of patients with right-sided tumor and anatomic vascular anomalies where the feeding artery originated from arteries other than the SMA (Supplementary Figure S4, available at https://doi.org/10.1016/j.esmogo.2025.100231). The optimal lymphadenectomy approach in such exceptional cases should be explored in future studies. Finally, long-term follow-up data are needed to accurately estimate the incidence of recurrence in unresected LNs within our population, thereby enhancing our understanding of *in vivo* lymphatic status in colon cancer. However, as previous multicenter research did not observe recurrence in unresected pericolic LNs located >10 cm from the primary tumor,^20^ and non-level C lymphadenectomy was carried out in only 24% of our study cohort, with almost half of these patients presenting with T1/T2 tumors, current findings regarding positivity of distant pericolic or central nodes are not expected to undergo significant changes.

In conclusion, this is the first large-scale, multi-national, prospective observational study for LN mapping with Western and Eastern colon cancer patients where the anatomical distribution of pericolic, intermediate, and central LNs were analyzed individually in terms of some essential perspectives including the location of the primary tumor and feeding arteries and tumor stage. This study demonstrated that there is no specific ethnic difference in the anatomical distribution of LNs and the primary feeding artery, which suggests that the extent of lymphadenectomy for colon cancer can be standardized internationally. Unnecessary longer bowel resection and shorter bowel resection that may jeopardize oncological curability should be avoided. In this regard, the ‘10-cm rule’ for the bowl-resection margin is a reasonable criterion because it ensures *en bloc* removal of pericolic LNs and lymphatic network system toward the root of the feeding artery that is at risk of harboring metastatic tumors. In addition, complete removal of intermediate LNs should be regarded as a routine surgical procedure in colon cancer surgery even for T1 or T2 tumors. The favorable short-term outcomes associated with level C radicality warrant the implementation of central LN dissection, though its indication should be tailored according to stage and location of the tumor, despite the current global trend toward extensive surgery.

## Supplementary data

Supplementary Material

Supplementary Protocol
