# Supplementary material for: Lymph node mapping-based optimal bowel-resection margin and central radicality in colon cancer surgery: an international, prospective, observational cohort study
Source: ESMO Gastrointest Oncol. 2025 Aug 27;9:100231. doi: 10.1016/j.esmogo.2025.100231 (PMC12836715; doi:10.1016/j.esmogo.2025.100231)
Supplement: Supplementary Material [file mmc1.pdf]

Supplementary materials

Table of contents

Supplementary Figures

Suppl. Figure 1	CONSORT diagram of the International Prospective Observational Cohort Study for Optimal Bowel Resection Extent and Central Radicality for Colon Cancer (T-REX study; ClinicalTrials.gov; NCT02938481)	Page 1
Suppl. Figure 2	Schema for the central radicality in the T-REX study	Page 2
Suppl. Figure 3	Pericolic lymph node status and the location of the primary feeding artery supplying the pericolic area	Page 3
Suppl. Figure 4	A case with cancer of the ascending colon and a feeding artery that originated from an artery other than the superior mesenteric artery	Page 4

Supplementary Tables

Suppl. Table 1	Institutions that participated in the International Prospective Observational Cohort Study for Optimal Bowel Resection Extent and Central Radicality for Colon Cancer (T-REX Study)	Page 5
Suppl. Table 2	Baseline characteristics of the study population	Page 6
Suppl. Table 3	Pericolic lymph node status stratified by the pathological T stage	Page 7
Suppl. Table 4	Location of the primary feeding artery supplying the pericolic region	Page 8
Suppl. Table 5	Anatomical location of the most distant metastatic pericolic lymph node stage	Page 9
Suppl. Table 6	Clinicopathological characteristics of patients with metastasis in pericolic lymph nodes located >10 cm from the primary tumor	Page 10
Suppl. Table 7	Central radicality stratified by the primary feeding artery and T stage	Page 11

Enrolled patients
N = 3,696
- Between 30 May 2013 and 31 December 2018
- 31 institutions from 6 countries (Japan, Korea, Russia, Lithuania, Germany, and Taiwan)

Inclusion Criteria
1. Histologically proven colon adenocarcinoma
2. Pathological Stage I, II, or III
3. Potentially curative surgery
4. Informed consent for observational data collection

Exclusion Criteria
1. Multiple colon cancers (excluding Tis)
2. Preoperative adjuvant therapy

Excluded (N = 49)

Duplicate registration (N = 7)

Patients who don't meet inclusion criteria (N = 7)
- No informed consent (1)
- No operation (2)
- Rectal cancer (4)

Patients who meet exclusion criteria (N = 1)
- Multiple colon cancers (1)

Patients with missing data on elemental items (N = 29)
- Number of retrieved lymph node (7)
- Tumor location (6)
- Central radicality (6)
- pT (5)
- Length of resected bowel (2)
- Central radicality and number of retrieved lymph node (2)
- Central radicality and tumor location (1)

Withdrawal of registration according to the request from institution (N = 5)

Analysis set
N = 3,647
(31 institutions, 6 countries)

Supplementary Figure 1. CONSORT diagram of the International Prospective Observational Cohort Study for Optimal Bowel Resection Extent and Central Radicality for Colon Cancer (T-REX study; ClinicalTrials.gov: NCT02938481)

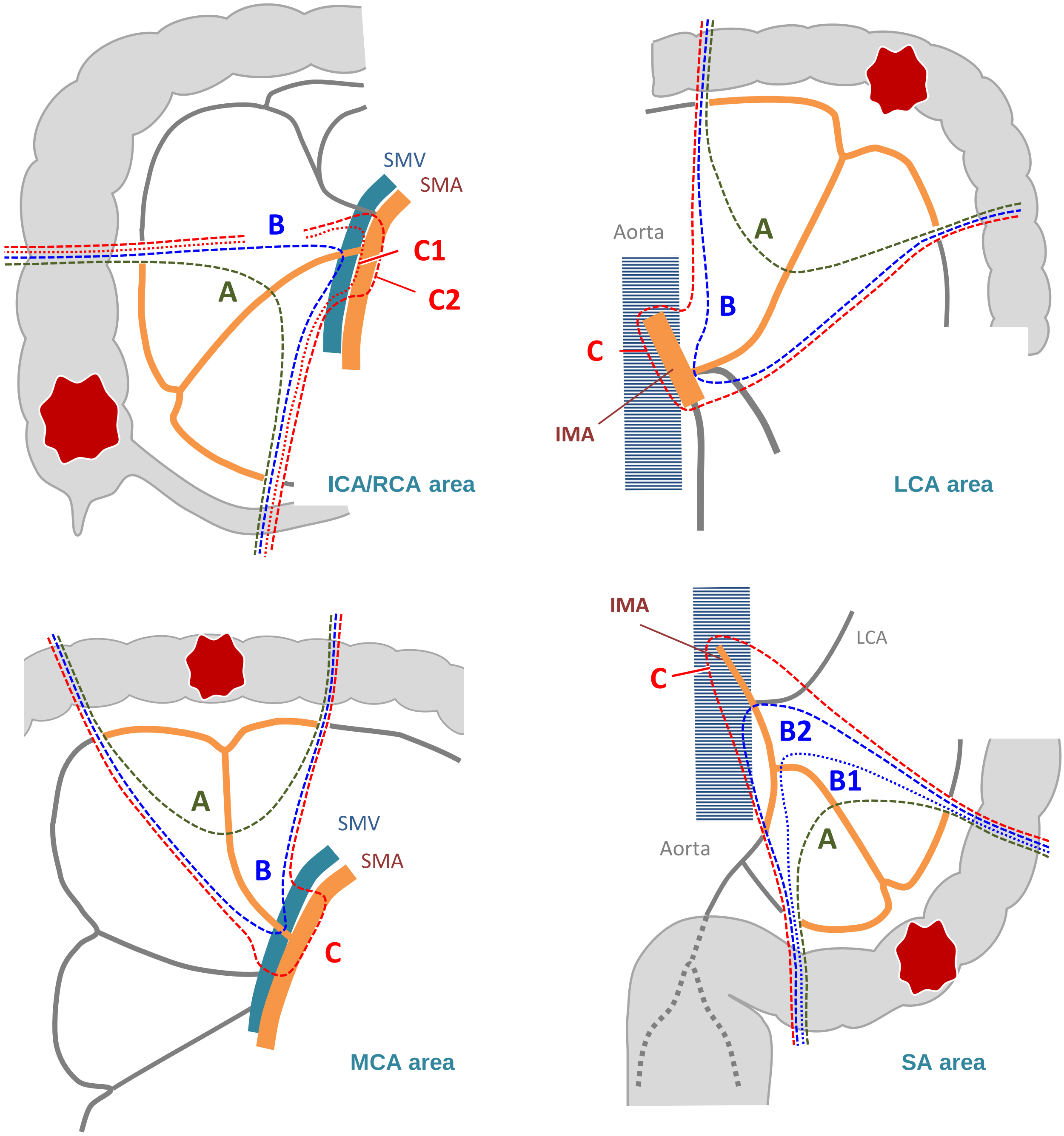

Supplementary Figure 2. Schema for the central radicality in the T-REX study

Area of the ileocolic artery (ICA), right colic artery (RCA), or middle colic artery (MCA)

Central radicality was recorded as Level A when the mesocolic excision was performed at the middle of the colic artery; as Level B when performed at the origin of the colic artery without exposure of the superior mesenteric vein (SMV); and Level C when central lymph nodes (LN) around the origin of the colic artery, including those along the SMV/superior mesenteric artery (SMA), were dissected in addition to Level B lymphadenectomy. A subclassification was provided for Level C for tumors in the ICA/RCA area based on whether the dissected central LNs were those along only the SMV (Level C1) or those along both the SMV and SMA (Level C2).

Area of the left colic artery (LCA) or sigmoid artery (SA)

Central radicality was recorded as Level A when mesocolic excision was performed at the middle of the colic artery; Level B when performed at the origin of the colic artery; and Level C when it was performed at the origin of the inferior mesenteric artery (IMA). A subclassification was provided for Level B for tumors in the SA area based on whether mesocoic excision was performed at the origin of the SA (Level B1) or at the origin of the LCA (Level B2).

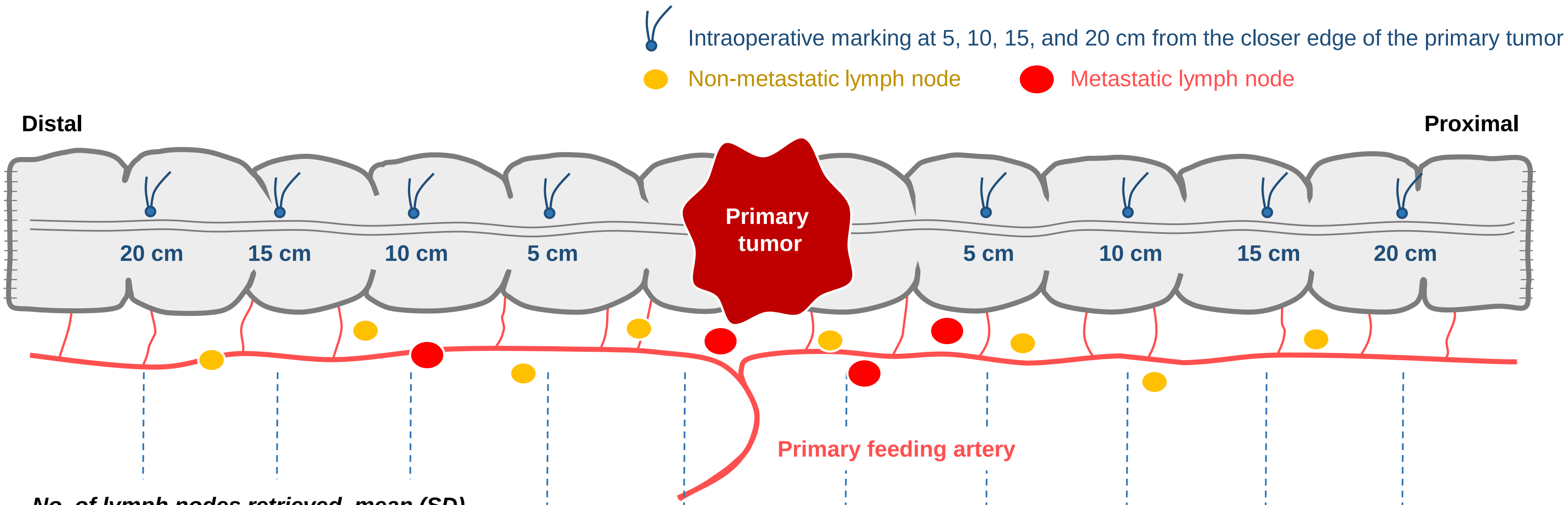

No. of lymph nodes retrieved, mean (SD)											
	0.1 (0.8)	0.1 (0.7)	0.4 (1.3)	2.5 (2.6)	4.3 (3.3)	5.2 (4.9)	4.3 (3.7)	2.9 (3.3)	0.7 (2.2)	0.2 (1.3)	0.1 (1.2)
No. of patients with lymph node metastasis (%)											
	0	0	2 (0.1)	41 (1)	318 (9)	987 (27)	390 (11)	55 (2)	4 (0.1)	1 (0.03)	0
Location of the primary feeding artery: No. of patients (%)											
	0	0	7 (0.2)	63 (2)	486 (13)	2117 (58)	812 (22)	133 (4)	4 (0.1)	0	0

Supplementary Figure 3. Pericolic lymph node status and the location of the primary feeding artery supplying the pericolic area

Both the number of the harvested lymph nodes and the incidence of metastasis were greatest in the primary tumor region and decreased with distance from the primary tumor. Both in proximal and distal sides, only 0.1% metastatic pericolic nodes were located >10 cm from the primary tumor, which is infrequent. Similarly, patients without a primary feeding artery supply to the pericolic region within 10 cm from the primary tumor were rare (0.3% of all participants).

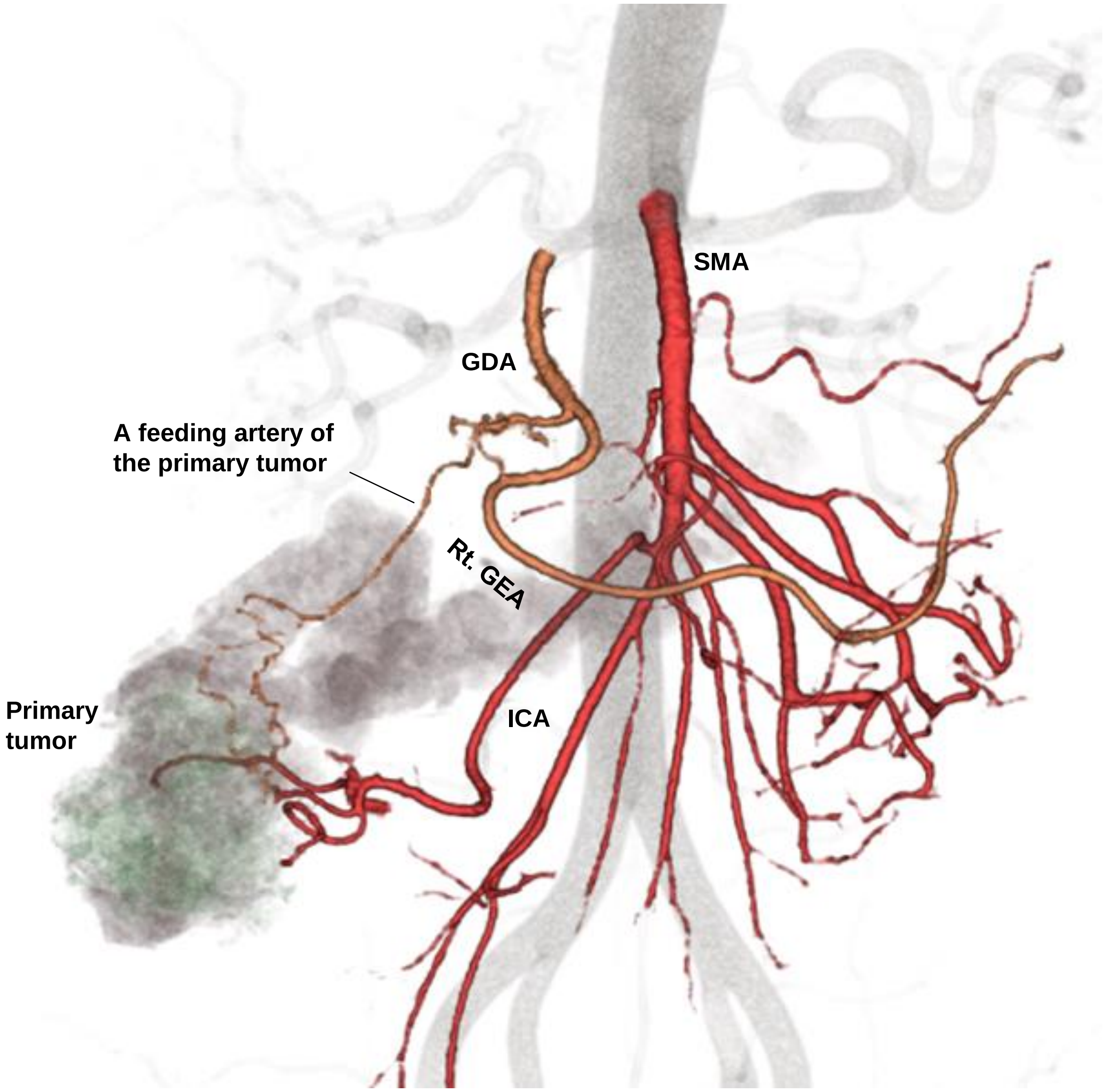

Supplementary Figure 4. A case with cancer of the ascending colon and a feeding artery that originated from an artery other than the superior mesenteric artery

In the T-REX study, central lymph nodes (LN) were defined as those along the superior mesenteric artery/vein (SMA/SMV) at the origin of the feeding artery in tumors in the right-sided colon. In rare instances, however, the primary feeding artery originates from arteries other than the SMA, and data on the extra-mesenteric LNs were not collected in this study. In the representative case, for example, extra-mesenteric LNs such as pancreatic head or epigastric LNs may be considered central LNs. Future studies to ascertain the clinical impact of the removal of extramesenteric LNs in cases with such anatomic vascular anomalies are warranted.

SMA, superior mesenteric artery; GDA, gastroduodenal artery; GEA, gastroepiploic artery; ICA, ileocolic artery

Supplementary Table 1. Institutions that participated in the International Prospective Observational Cohort Study for Optimal Bowel Resection Extent and Central Radicality for Colon Cancer (T-REX Study)

	Institution (country)
1.	Aichi Cancer Centre Hospital (Japan)
2.	Asan Medical Center (Republic of Korea)
3.	First Moscow State Medical University (Russia)
4.	Kanagawa Cancer Centre Hospital (Japan)
5.	Keiyukai Sapporo Hospital (Japan)
6.	Klaipeda University Hospital (Lithuania)
7.	Kurume University School of Medicine (Japan)
8.	Kyorin University School of Medicine (Japan)
9.	Mie University Graduate School of Medicine (Japan)
10.	National Cancer Centre Hospital East (Japan)
11.	National Cancer Centre Central Hospital (Japan)
12.	National Cancer Institute (Lithuania)
13.	National Defense Medical College (Japan)
14.	National Taiwan University Hospital (Taiwan)
15.	Niigata Cancer Center Hospital (Japan)
16.	Osaka International Cancer Institute (Japan)
17.	Saisei-kai Yokohama-shi Nanbu Hospital (Japan)
18.	Shiga University of Medical Science (Japan)
19.	Shizuoka Cancer Centre Hospital (Japan)
20.	Takano Hospital (Japan)
21.	Teikyo University Chiba Medical Center (Japan)
22.	Teikyo University School of Medicine (Japan)
23.	Tochigi Cancer Center (Japan)
24.	Tokyo Medical and Dental University (Japan)
25.	Tokyo Women's Medical University (Japan)
26.	University Hospital Erlangen (Germany)
27.	Wakayama Medical University (Japan)
28.	Yamagata Prefectural Central Hospital (Japan)
29.	Yokohama City University (Japan)
30.	Yokohama City University Medical Center (Japan)
31.	Yonsei University (Republic of Korea)

Supplementary Table 2. Baseline characteristics of the study population

	All (n=3647)	Japan (n=2797)	Korea (n=450)	Russia (n=149)	Lithuania (n=121)	Germany (n=90)	Taiwan (n=40)
Age, years, Mean (SD)	68 (12)	69 (11)	62 (12)	63 (12)	66 (11)	66 (14)	69 (12)
Sex, No.(%)							
Male	1858 (51)	1445 (52)	227 (50)	62 (42)	53 (44)	55 (61)	16 (40)
Female	1789 (49)	1352 (48)	223 (50)	87 (58)	68 (56)	35 (39)	24 (60)
Height, cm, Mean (SD)	161 (10)	159 (9)	161 (9)	168 (9)	168 (8)	171 (10)	158 (9)
Body weight, kg, Mean (SD)	60 (14)	58 (12)	62 (11)	78 (18)	81 (17)	78 (20)	62 (15)
Body mass index, Mean (SD)	23 (4)	23 (4)	24 (3)	27 (5)	29 (5)	27 (6)	25 (5)
Surgical approach, No. (%)							
Open	890 (24)	652 (23)	16 (4)	73 (49)	84 (69)	65 (72)	0
Laparoscopic	2757 (76)	2145 (77)	434 (96)	76 (51)	37 (31)	25 (28)	40 (100)
Central radicality*, No. (%)							
A	191 (5)	175 (6)	8 (2)	0	7 (6)	1 (1)	0
B	689 (19)	516 (18)	105 (23)	5 (3)	25 (21)	38 (42)	0
C	2767 (76)	2106 (75)	337 (75)	144 (97)	89 (74)	51 (57)	40 (100)
Pathological T stage, No. (%)							
T1	660 (18)	561 (20)	69 (15)	7 (5)	13 (11)	7 (8)	3 (8)
T2	534 (15)	406 (15)	70 (16)	14 (9)	14 (12)	20 (22)	10 (25)
T3	1867 (51)	1337 (48)	255 (57)	113 (76)	80 (66)	57 (63)	25 (63)
T4	586 (16)	493 (18)	56 (12)	15 (10)	14 (12)	6 (7)	2 (5)
No. of LN retrieved, Mean (SD)							
All	32 (16)	31 (15)	30 (17)	51 (25)	29 (14)	30 (14)	32 (11)
Pericolic	21 (11)	21 (11)	18 (11)	30 (15)	23 (12)	19 (11)	18 (8)
Intermediate	6 (5)	6 (5)	5 (4)	12 (9)	4 (3)	8 (6)	11 (4)
Main	5 (5)	5 (4)	8 (9)	9 (8)	3 (2)	2 (3)	3 (2)
No. of LN involved, No. (%)							
0	2264 (62)	1728 (62)	284 (63)	87 (58)	79 (65)	61 (68)	25 (63)
1–3	983 (27)	778 (28)	113 (25)	33 (22)	28 (23)	20 (22)	11 (28)
≥4	400 (11)	291 (10)	53 (12)	29 (20)	14 (12)	9 (10)	4 (10)
LN ratio† (%)	4	4	5	4	4	4	5
Tumor grade, No. (%)							
G1	1311 (36)	1159 (41)	51 (11)	71 (48)	9 (7)	0	21 (53)
G2	2044 (56)	1454 (52)	374 (83)	64 (43)	94 (78)	41 (46)	17 (43)
G3	261 (7)	158 (6)	20 (5)	14 (9)	18 (15)	49 (54)	2 (5)
G4	30 (1)	26 (1)	4 (1)	0	0	0	0
Tumor location [1]‡, No. (%)							
C	470 (13)	366 (13)	35 (8)	23 (15)	18 (15)	14 (16)	14 (35)
A	1115 (31)	884 (32)	133 (30)	31 (21)	25 (21)	26 (29)	16 (40)
T	539 (15)	431 (15)	48 (11)	13 (9)	16 (13)	22 (24)	9 (23)
D	244 (7)	174 (6)	30 (7)	16 (11)	17 (14)	6 (7)	1 (3)
S	1279 (35)	942 (34)	204 (45)	66 (44)	45 (37)	22 (24)	0
Tumor location [2] , No. (%)							
Hepatic flexure	364 (10)	295 (11)	37 (8)	6 (4)	10 (8)	9 (10)	7 (18)
Splenic flexure	192 (5)	155 (6)	8 (2)	6 (4)	16 (13)	6 (7)	1 (3)
Non-flexure	3091 (85)	2347 (84)	405 (90)	137 (92)	95 (79)	75 (83)	32 (80)
Primary feeding artery§, No. (%)							
ICA	1335 (37)	1052 (38)	133 (30)	52 (35)	42 (35)	37 (41)	19 (48)
RCA	170 (5)	131 (5)	21 (5)	1 (1)	4 (3)	1 (1)	12 (30)
MCA	594 (16)	480 (17)	61 (14)	16 (11)	7 (6)	22 (24)	8 (20)
LCA	248 (7)	173 (6)	30 (47)	14 (9)	25 (21)	5 (6)	1 (3)
SA	1300 (36)	961 (34)	205 (46)	66 (44)	43 (36)	25 (28)	0
Bowel length, cm, Mean (SD)							
Proximal	13 (10)	13 (10)	11 (7)	16 (14)	13 (5)	16 (8)	25 (13)
Distal	12 (8)	12 (8)	10 (7)	14 (8)	13 (6)	14 (7)	21 (12)

LN=lymph node. SD=standard deviation. *The level of central radicality was recorded with the original categorization of levels A to C according to pre-specified anatomical landmarks (Supplementary Fig.2). †The ratio between the metastatic and the removed lymph nodes. ‡C, A, T, D, and S represent the cecum, ascending colon, transverse colon, descending colon, and sigmoid colon, respectively. §ICA, RCA, MCA, LCA, and SA represent the ileocolic artery, right colic artery, middle colic artery, left colic artery, and sigmoid artery, respectively.

Supplementary Table 3. Pericolic lymph node status stratified by the pathological T stage

	Overall	Pericolic segments subdivided by the distance from the primary tumor*, cm					
		Primary tumor region	0 < *D* ≤ 5	5 < *D* ≤ 10	10 < *D* ≤ 15	15 < *D* ≤ 20	*D* > 20
**No. of retrieved pericolic lymph nodes, Mean (SD)**							
All (n=3647)	21 (11)	5 (5)	9 (5)	5 (5)	1 (3)	0.3 (1)	0.2 (2)
T1 (n=660)	17 (10)	3 (3)	8 (5)	5 (5)	0.8 (2)	0.1 (1)	0.1 (1)
T2 (n=534)	19 (10)	5 (4)	8 (5)	5 (5)	1 (3)	0.2 (1)	0.1 (1)
T3 (n=1867)	22 (11)	6 (5)	9 (5)	5 (5)	1 (3)	0.3 (2)	0.2 (2)
T4 (n=586)	24 (13)	7 (5)	10 (6)	6 (5)	1 (3)	0.2 (1)	0.2 (2)
**Patients with metastatic pericolic lymph nodes, No. (%)**							
All (n=3647)	1281 (35)	987 (27)	596 (16)	92 (3)	6 (0.2)	1 (0.03)	0
T1 (n=660)	74 (11)	39 (6)	35 (5)	5 (0.8)	0	0	0
T2 (n=534)	120 (23)	78 (15)	67 (13)	12 (2)	1 (0.2)	0	0
T3 (n=1867)	718 (38)	560 (30)	311 (17)	48 (3)	4 (0.2)	1 (0.1)	0
T4 (n=586)	369 (63)	310 (53)	183 (31)	27 (5)	1 (0.2)	0	0

D=Distance from the closest primary tumor edge. SD=Standard deviation.

*Sum of the number of lymph nodes (upper row) and patient (lower row) both proximal and distal to the primary tumor

Supplementary Table 4. Location of the primary feeding artery supplying the pericolic region

	**Pericolic segments subdivided by the distance from the primary tumor (*D*), cm**					**P value**
	**Primary tumor region**	**0 < *D* ≤ 5**	**5 < *D* ≤ 10**	**10 < *D* ≤ 15**	***D* >15**	
All, No. (%)	2117 (58)	1298 (36)	196 (5)	11 (0.3)	0	<0.0001
Tumor location [1]*, No. (%)						
C (n=463)	373 (81)	85 (18)	4 (1)	1 (0.2)	0	<0.0001
A (n=1110)	557 (50)	484 (44)	67 (6)	2 (0.2)	0	
T (n=534)	264 (49)	211 (40)	54 (10)	5 (0.9)	0	
D (n=243)	135 (56)	89 (37)	17 (7)	2 (0.8)	0	
S (n=1272)	788 (62)	429 (34)	54 (4)	1 (0.1)	0	
Tumor location [2], No. (%)						
Non-flexure site (n=3066)	1852 (60)	1062 (35)	148 (5)	4 (0.1)	0	<0.0001
Hepatic flexure (n=364)	176 (48)	157 (43)	28 (8)	3 (0.8)	0	
Splenic flexure (n=192)	89 (46)	79 (41)	20 (10)	4 (2)	0	
Primary feeding artery†, No. (%)						
ICA (n=1,323)	819 (62)	448 (34)	54 (4)	2 (0.2)	0	<0.0001
RCA (n=170)	94 (55)	67 (39)	7 (4)	2 (1)	0	
MCA (n=590)	271 (46)	259 (44)	56 (10)	4 (0.7)	0	
LCA (n=246)	132 (54)	87 (35)	25 (10)	2 (0.8)	0	
SA (n=1,293)	801 (62)	437 (34)	54 (4)	1 (0.1)	0	
Countries, No. (%)						
Japan (n=2796)	1736 (62)	976 (35)	78 (3)	6 (0.2)	0	<0.0001
Korea (n=450)	202 (45)	176 (39)	68 (15)	4 (0.9)	0	
Russia (n=149)	61 (41)	76 (51)	12 (8)	0	0	
Lithuania (n=121)	76 (63)	25 (21)	20 (17)	0	0	
Germany (n=66)	23 (35)	30 (46)	12 (18)	1 (2)	0	
Taiwan (n=40)	19 (48)	15 (38)	6 (15)	0	0	

*C, A, T, D, and S represent the cecum, ascending colon, transverse colon, descending colon, and sigmoid colon, respectively. †Data from a total of 3622 patients in the analysis dataset were analyzed after excluding 25 patients whose data on the location of the primary feeding artery were missing. ICA, RCA, MCA, LCA and SA represent the ileocolic artery, right colic artery, middle colic artery, left colic artery, and sigmoid artery

Supplementary Table 5. Anatomical location of the most distant metastatic pericolic lymph node

	Pericolic segment harboring the most distant metastatic pericolic LN							
	**No metastasis in the pericolic LNs**	**Primary tumor region**	**0 < *D* ≤ 5**	**5 < *D* ≤ 10**	**10 < *D* ≤ 15**	**15 < *D* ≤ 20**	***D* > 20**	***P* value**
Tumor location [1]*, No. (%)								0.39
C (n=470)	305 (65)	90 (19)	66 (14)	8 (2)	1 (0.2)	0	0	
A (n=1115)	724 (65)	190 (17)	164 (15)	35 (3)	1 (0.1)	1 (0.1)	0	
T (n=539)	376 (70)	86 (16)	67 (12)	10 (2)	0	0	0	
D (n=244)	153 (63)	43 (18)	45 (18)	3 (1)	0	0	0	
S (n=1279)	808 (63)	239 (19)	193 (15)	35 (3)	4 (0.3)	0	0	
Tumor location [2], No. (%)								0.59
Non-flexure site (n=3091)	1985 (64)	556 (18)	460 (15)	83 (3)	6 (0.2)	1 (0.0)	0	
Hepatic flexure (n=364)	253 (70)	62 (17)	45 (12)	4 (1)	0	0	0	
Splenic flexure (n=192)	128 (67)	30 (16)	30 (16)	4 (2)	0	0	0	
Location of the primary feeding artery, No. (%)								0.28
Primary tumor region (n=2117)	1356 (64)	372 (18)	328 (16)	59 (3)	2 (0.1)	0	0	
D ≤ 5 (n=1298)	842 (65)	245 (19)	179 (14)	27 (2)	4 (0.3)	1 (0.1)	0	
5 < *D* ≤ 10 (n=196)	142 (72)	23 (12)	26 (13)	5 (3)	0	0	0	
10 < *D* (n=11)	6 (55)	4 (36)	1 (9)	0	0	0	0	
Country, No. (%)								<0.0001
Japan (n=2797)	1800 (64)	510 (18)	412 (15)	70 (3)	5 (0.2)	0	0	
Korea (n=450)	305 (68)	40 (9)	86 (19)	18 (4)	1 (0.2)	0	0	
Russia (n=149)	91 (61)	43 (29)	14 (9)	0	0	1 (0.7)	0	
Lithuania (n=121)	81 (67)	22 (18)	16 (13)	2 (2)	0	0	0	
Germany (n=90)	63 (70)	19 (21)	7 (8)	1 (1)	0	0	0	
Taiwan (n=40)	26 (65)	14 (35)	0	0	0	0	0	

LN, lymph node; *D*, distance from the closest primary tumor edge (cm); *C, A, T, D, and S represent the cecum, ascending colon, transverse colon, descending colon, and sigmoid colon, respectively; †data from a total of 3622 patients in the analysis dataset were analyzed after excluding 25 patients whose data on the location of the primary feeding artery were missing.

Supplementary Table 6. Patients with metastasis in pericolic lymph nodes located >10 cm from the primary tumor

Case	Country	Sex (age, years)	Tumor location*	Tumor grade	T stage	Location of the primary feeding artery	Location of the most distant metastatic LN	No. of harvested LNs	No. of metastatic LNs		
									Pericolic	Intermediate	Central
1	Japan	Male (75)	S	G1	pT3	Distal to the primary tumor (≤5 cm)	10 < *D* ≤ 15	26	7	3	0
2	Japan	Female (61)	S	G1	pT2	Proximal to the primary tumor (≤5 cm)	10 < *D* ≤ 15	21	2	0	0
3	Japan	Female (69)	S	G1	pT3	Proximal to the primary tumor (≤5 cm)	10 < *D* ≤ 15	17	3	0	0
4	Japan	Male (45)	C	G2	pT4a	Primary tumor region	10 < *D* ≤ 15	18	3	3	1
5	Japan	Female (77)	A	G2	pT3	Primary tumor region	10 < *D* ≤ 15	25	7	2	2
6	Korea	Female (81)	S	G2	pT3	Distal to the primary tumor (≤5cm)	10 < *D* ≤ 15	24	1	0	1
7	Russia	Female (52)	A	G2	pT3	Proximal to the primary tumor (≤5 cm)	15 < *D* ≤ 20	62	9	0	0

*C: Cecum, A: Ascending colon (non-flexural site), S: Sigmoid colon, LN: Lymph node; *D*, distance from the closest primary tumor edge (cm).

Supplementary Table 7. Central radicality stratified by the primary feeding artery and T stage

Primary feeding artery*	pT	Central radicality†, No. (%)							*P* value
		A	B	B1	B2	C	C1	C2	
ICA/RCA	T1 (n=249)	14 (6)	63 (25)	–	–	–	116 (47)	56 (23)	<0.0001
	T2 (n=232)	6 (3)	32 (14)	–	–	–	129 (56)	65 (28)	
	T3 (n=775)	37 (5)	85 (11)	–	–	–	411 (53)	242 (31)	
	T4 (n=249)	8 (3)	19 (8)	–	–	–	149 (60)	73 (29)	
MCA	T1 (n=105)	16 (15)	63 (60)	–	–	26 (25)	–	–	<0.0001
	T2 (n=79)	2 (3)	26 (33)	–	–	51 (65)	–	–	
	T3 (n=329)	20 (6)	99 (30)	–	–	210 (64)	–	–	
	T4 (n=81)	8 (10)	18 (22)	–	–	55 (68)	–	–	
LCA	T1 (n=41)	2 (5)	22 (54)	–	–	17 (42)	–	–	0.0015
	T2 (n=25)	3 (12)	5 (20)	–	–	17 (68)	–	–	
	T3 (n=127)	6 (5)	28 (22)	–	–	93 (73)	–	–	
	T4 (n=55)	2 (4)	11 (20)	–	–	42 (76)	–	–	
SA	T1 (n=265)	8 (3)	–	10 (4)	80 (30)	167 (63)	–	–	<0.0001
	T2 (n=198)	13 (7)	–	5 (3)	27 (14)	153 (77)	–	–	
	T3 (n=636)	38 (6)	–	26 (4)	53 (8)	519 (82)	–	–	
	T4 (n=201)	8 (4)	–	4 (2)	13 (7)	176 (88)	–	–	

*ICA, RCA, MCA, LCA, and SA represent the ileocolic artery, right colic artery, middle colic artery, left colic artery, and sigmoid artery, respectively; †the level of central radicality was recorded with the original categorization of levels A to C according to pre-specified anatomical landmarks (Supplementary Figure 2).
